# Phase II study of nab-paclitaxel with gemcitabine for relapsed/refractory small cell lung cancer

**DOI:** 10.3389/fonc.2024.1303268

**Published:** 2024-07-31

**Authors:** Margaret M. Byrne, Grerk Sutamtewagul, William Zeitler, Sarah L. Mott, Gideon K.D. Zamba, Arsenije Kojadinovic, Jun Zhang, Taher Abu-Hejleh, Gerald Clamon, Muhammad Furqan

**Affiliations:** ^1^ Division of Hematology, Oncology, and Blood & Marrow Transplantation, Department of Internal Medicine, University of Iowa, Iowa City, IA, United States; ^2^ Holden Comprehensive Cancer Center, University of Iowa, Iowa City, IA, United States; ^3^ Department of Biostatistics, University of Iowa, Iowa City, IA, United States

**Keywords:** small cell lung cancer, gemcitabine, nab-paclitaxel, clinical trial, single-arm

## Abstract

**Background:**

Patients with small cell lung cancer (SCLC) often respond to first-line chemoimmunotherapy. However, relapse is inevitable and is associated with a poor prognosis. Treatments for relapsed SCLC, such as lurbinectedin and topotecan, are limited by modest efficacy and significant hematologic adverse events, leaving a need for newer therapeutic agents or regimens. The combination of gemcitabine and nab-paclitaxel is active and safe in other types of malignancies, such as pancreatic cancer.

**Patients and methods:**

We conducted a phase II trial evaluating the efficacy and safety of gemcitabine and nab-paclitaxel in patients with relapsed/refractory SCLC. The primary endpoint was objective response rate (ORR), defined as the proportion of patients with confirmed complete or partial response. Secondary endpoints included time to progression (TTP), progression-free survival (PFS), overall survival (OS), and safety.

**Results:**

Between October 2016 and May 2021, 32 patients were enrolled. Patients were followed for a median of 9.3 months (range 1.8–65.2). Median age was 65 years (range 48–81). Fifty percent of patients were female. Fifty-three percent of patients had platinum-resistant/refractory relapsed SCLC. The ORR was 28.1% (95% confidence interval [CI] 15.5–100%). Median PFS was 2.9 months (95% CI 2.4–3.6), and median OS was 9.3 months (95% CI 5.2–12.4). Seven patients (21.9%) developed grade 3 or 4 neutropenia.

**Conclusion:**

Our study showed that the combination of gemcitabine and nab-paclitaxel led to encouraging outcomes in relapsed/refractory SCLC. Further studies are needed to compare this combination with other treatments used for relapsed SCLC, including lurbinectedin, temozolomide, and topotecan.

**Clinical trial registration:**

https://clinicaltrials.gov/study/NCT02769832?cond=NCT02769832&rank=1, identifier NCT02769832.

## Introduction

Among the estimated 130,000 lung cancer deaths in 2023, approximately 15% will be due to small cell lung cancer (SCLC) (1). Nearly two-thirds of patients with SCLC will present with cancer that has metastasized beyond the thoracic cavity, known as extensive stage small cell lung cancer (ES-SCLC) (1, 2). For several decades, standard front-line therapy for patients with ES-SCLC has been platinum chemotherapy with etoposide (3). Recently, the addition of an immune checkpoint inhibitor (ICI), atezolizumab or durvalumab, to standard chemotherapy has shown a modest improvement in overall survival in patients with ES-SCLC (4–6). While ES-SCLC is initially sensitive to this combination of chemo-immunotherapy, responses are not durable, and almost all patients inevitably develop disease progression (1).

Effective therapies for patients with relapsed SCLC remain limited, particularly for those with resistant or refractory disease, defined as disease progression within 90 days of chemotherapy or while on chemotherapy (7–9). Since 1996, the primary treatment for patients with relapsed SCLC was topotecan, although it causes significant hematologic side effects (10). In 2020, lurbinectedin was the first treatment in many years to show promise in relapsed SCLC with an improvement in response rate to 35% and relatively fewer hematologic adverse events (11). However, a phase III study of lurbinectedin with doxorubicin failed to meet its primary endpoint of improved overall survival (OS) in patients with relapsed SCLC compared to the investigator’s choice of topotecan or the combination of cyclophosphamide, doxorubicin, or vincristine (CAV) (12). In addition, although lurbinectedin led to less hematologic toxicity than topotecan, grade 3 or 4 neutropenia developed in over 40% of patients (11). Studies evaluating single-agent chemotherapy for relapsed SCLC have demonstrated modest efficacy. Finally, with expanding indications for ICIs into front-line treatment, the benefit from ICIs in second or third-line is unclear, leaving a paucity of safe and effective treatment options for patients with relapsed SCLC.

Both gemcitabine and paclitaxel are active in relapsed SCLC, although response rates to these agents individually are less than optimal (13–16). Nab-paclitaxel is an albumin-bound formulation of paclitaxel, created to decrease the rate of infusion reactions and potential side effects (17). Gemcitabine and nab-paclitaxel have distinct mechanisms of action and have been shown to have additive/synergistic activity and are relatively safe in the treatment of other types of malignancies, such as pancreatic cancer (18). In addition, in a phase I study, this combination showed potential activity in previously treated SCLC (19). We report a phase II clinical trial evaluating gemcitabine and nab-paclitaxel combination in patients with relapsed SCLC (NCT02303977).

## Materials and methods

### Study design

We conducted an open-label, single-arm, phase II study to evaluate nab-paclitaxel and gemcitabine in patients with relapsed SCLC. Eligible participants were adults (≥18 years) with histologically or cytologically confirmed SCLC, Eastern Cooperative Oncology Group (ECOG) performance score 0–2, at least 1 measurable lesion as defined by Response Evaluation Criteria in Solid Tumors (RECIST v1.1), disease progression during or after first-line chemotherapy, including progression after chemoradiation for limited stage disease if progressed within 12 months of treatment, adequate hematologic function (ANC ≥1800/mm^3^, platelet count ≥100,000/mm^3^, and hemoglobin ≥9.0), hepatic function (bilirubin ≤1.5 x ULN, AST and ALT ≤2.5 x ULN or AST and ALT ≤5 x ULN if liver metastases were present), and renal function (serum creatinine ≤ 1.5 x ULN). Prior treatment with ICIs, either with first-line chemotherapy or as second-line therapy, was allowed after a protocol modification when these agents got the FDA approval.

Key exclusion criteria included previous receipt of a taxane, history of other invasive malignancy in the past 12 months, pre-existing peripheral neuropathy (grade ≥2 according to Common Terminology Criteria for Adverse Events version 4.03 (CTCAEv4.03), serious medical condition in the previous 6 months, or untreated brain metastases requiring radiation, surgery, or continued use of steroids. Treated brain metastases were required to be stable for at least 4 weeks and steroids were to be discontinued for at least 7 days before study therapy.

### Study oversight

The study was performed at the University of Iowa Holden Cancer Center (HCCC). It was approved by the institutional review board (HawkIRB 201512799) and was performed per the Declaration of Helsinki and Good Clinical Practice guidelines. All patients provided written informed consent. The HCCC Data Safety and Monitoring Committee provided study oversight.

### Study treatment

Eligible patients received gemcitabine 1000 mg/m^2^ and nab-paclitaxel 100 mg/m^2^ on days 1 and 8 of a 21-day cycle. This was continued until disease progression or intolerable toxicity or withdrawal of consent. Dose modifications were allowed for low absolute neutrophil (ANC) and platelet counts. Dose delays due to toxicities, febrile neutropenia, and other illnesses were allowed for up to 3 weeks.

### Study objectives and endpoints

The primary endpoint of the study was to evaluate the objective response rate (ORR) according to RECIST version 1.1. Patients who achieved a partial or complete response underwent a confirmatory tumor assessment at least 4 weeks following the initial imaging demonstrating the response. Tumor assessments occurred at baseline and every 6 weeks while in the study. Secondary endpoints included time to progression (TTP), progression-free survival (PFS), OS, and safety. Adverse events (AEs) were graded using CTCAE v4.03.

### Statistical considerations and analyses

The primary objective of this phase II trial was to evaluate the anti-tumor activity of nab-paclitaxel and gemcitabine by testing the null hypothesis that the best ORR is less than 15% versus the alternative that it is greater (20). Best response was defined as a confirmed complete or partial response. The trial was conducted as a single-stage design having 80% power to detect a response rate of 35% with one-sided statistical testing performed at the 5% level of significance and assuming 12.5% lost to follow-up.

Primary statistical analysis focused on the best objective response rate estimated as a binomial proportion along with a one-sided 95% confidence interval (CI). Secondary analyses focused on TTP, PFS, OS, and safety. TTP was defined as the time from treatment initiation to the date of first documentation of disease progression. PFS was defined as the time from treatment initiation to the date of first documentation of disease progression or death due to any cause. Patients were censored at the date of the last radiographic assessment for progression. OS was defined as the time from treatment initiation to death due to any cause. Patients still alive were censored at the last date known to be alive. Survival probabilities were estimated and plotted using the Kaplan-Meier method. Estimates along with 95% pointwise CIs are reported. Incidence of adverse events attributable to the study drugs was graded, with the most severe grade per patient being reported.

## Results

### Patient population

**Figure 1:**
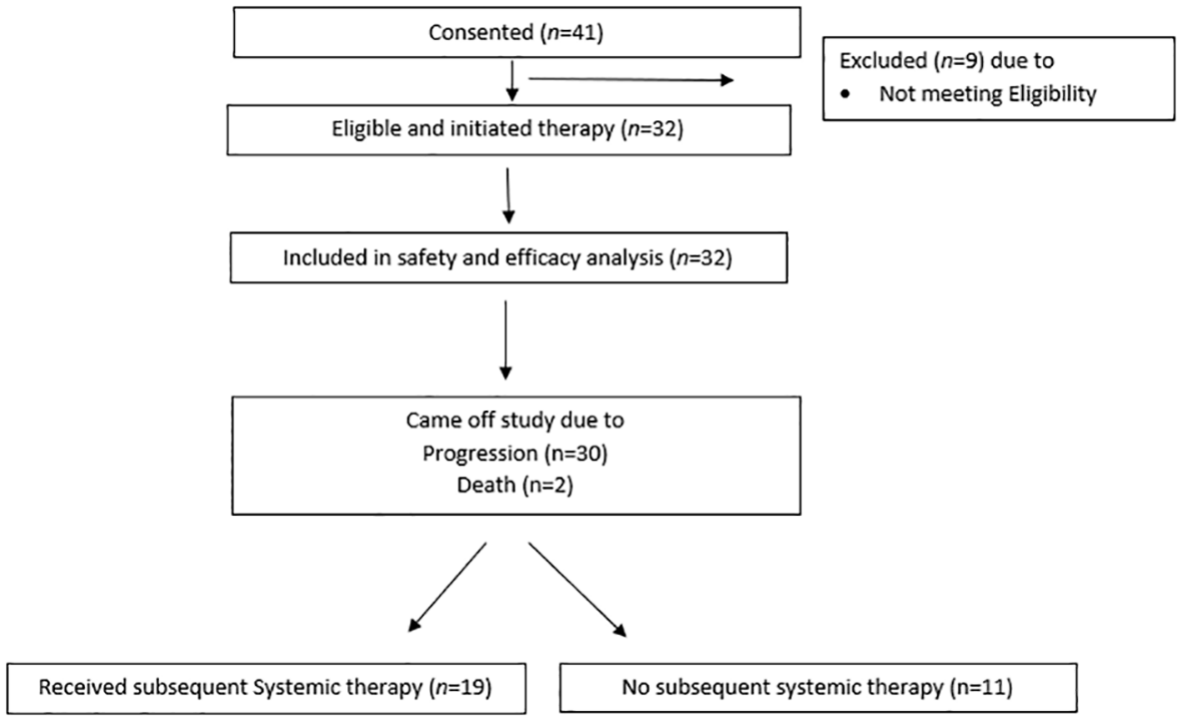
Consort diagram.

Between October 2016 and May 2021, 32 patients were enrolled (**Figure 1**). Median follow-up was 9.3 months (range 1.8–65.2). Fifty percent of the patients were male, and all patients were White (**Table 1**). The median age was 65 years (range 48–81). There were 12.5% of patients with an Eastern Cooperative Oncology Group (ECOG) performance score of 2. At diagnosis, 87.5% of patients had extensive disease. At the time of enrollment, patients had a median of 3 sites of disease involvement (range 1–5), 50% had bone metastases, and about 40% had treated brain metastases. Half of the patients had previously received an ICI, and over 60% had previously received radiation therapy to the thoracic cavity. Fifty-three percent of patients had platinum-resistant/refractory disease.

**Table 1:** Study patient characteristics.

Patient characteristics (n=32)	n (%)
Age, median (range)	65 (48–81)
Sex	
Female	16 (50.0)
Male	16 (50.0)
Race	
White	32 (100.0)
Ethnicity	
Non-Hispanic	32 (100.0)
ECOG performance score	
0	1 (3.1)
1	27 (84.4)
2	4 (12.5)
Stage at diagnosis	
Limited	4 (12.5)
Extensive	28 (87.5)
Number of sites involved, median (range)	3 (1–5)
Presence of brain metastases	13 (40.6)
Time since platinum chemotherapy	
< 3 months	17 (53.1)
3–6 months	9 (28.1)
> 6 months	6 (18.8)
Prior chest radiation	20 (62.5)
Prior immune therapy	18 (56.3)

ECOG, Eastern Cooperative Oncology Group.

### Treatment

Patients received a median of 4.0 cycles (range 2.0–13.0) of chemotherapy. In total, 46.9% of patients required a level 1 dose reduction (defined as gemcitabine 800 mg/m^2^ or nab-paclitaxel 80 mg/m^2^), and 40.6% required a level 2 dose reduction (defined as gemcitabine 600 mg/m^2^ or nab-paclitaxel 60 mg/m^2^), most commonly due to bone marrow toxicity. Twenty patients (62.5%) received growth factor support. Median dose received on treatment days for gemcitabine was 835.7 mg/m^2^ and 84.8 mg/m^2^ for abraxane.

### Efficacy

**Figure 2:**
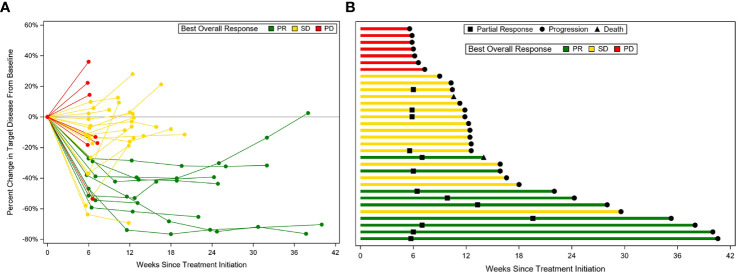
Response to treatment. Spider’s plot **(A)**; Swimmer’s plot **(B)**.

**Figure 3:**
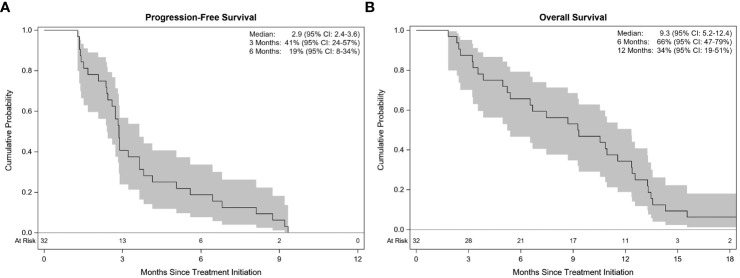
Kaplan-Meier estimates with 95% confidence. Progression-free survival **(A)** and Overall survival **(B)**.

All patients were included in the efficacy analysis. ORR was 28.1%, demonstrating a statistically significant increase compared to a historical control of 15.0% (p=0.04; **Figure 2**). ORR was 33.3% in patients with platinum-sensitive disease and 23.5% in patients with platinum-resistant/refractory disease. Fifty percent of patients (n=16) had stable disease, of which 25.0% had an unconfirmed PR (n=4). The disease control rate (DCR) was 78.1%. Thirty patients had disease progression during the follow-up period, while 2 patients died before progression. Median TTP was 2.9 months (95% CI 2.4–3.8), and median PFS was 2.9 months (95% CI 2.4–3.6; **Figure 3**). Median PFS was 2.8 months (95% CI 1.5–5.6) in patients with platinum-sensitive disease, and 2.9 months (95% CI 1.7–3.6) in patients with platinum-resistant/refractory disease. Median OS was 9.3 months (95% CI 5.2–12.4; **Figure 3**). In patients with platinum-sensitive disease, median overall survival was 10.8 months (95% CI 3.3–12.6), and median OS was 6.7 months (95% CI 3.5–12.4) in patients with platinum-resistant/refractory disease. Overall survival at 9, 12, and 18 months was 53%, 34%, and 6%, respectively. At 18 months, 2 patients (6.2%) were still alive; one patient (3.1%) was still alive at the time of data cut-off for the trial. Outcomes were not different with regards to presence or absence of baseline brain metastases

### Safety

All patients developed a treatment-related adverse event (**Table 2**). The most common adverse events included fatigue, hematologic events, gastrointestinal complaints, neuropathy, pneumonitis, loss of appetite/weight loss, changes in electrolytes and/or liver function tests, and fever. The most common grade 3 or higher adverse events were hematologic. Grade 3 or 4 neutropenia occurred in 21.9% of patients (n=7). Pneumonitis occurred in 18.8% of patients (n=6); one was grade 1 (3.1%), four were grade 2 (12.5%), and one was grade 5 (3.1%; **Table 3**). Of the 5 patients who developed grade 1 or 2 pneumonitis, 4 patients were treated with steroids, all of whom had resolution of pneumonitis. The patient with grade 5 pneumonitis had received radiation to the thoracic cavity and an ICI before enrollment on the trial. This patient was admitted to another facility with shortness of breath and was found to have concurrent disease progression. Hence, subject decided to pursue hospice, and pneumonitis remained untreated. Treatment discontinuation occurred in 6.3% of patients (n=2) due to grade 2 pneumonitis and patient preference.

**Table 2:** Treatment-related adverse events occurred with a frequency of ≥5%.

Toxicity	Grade					Total
	1	2	3	4	5	
Any toxicity	1 (3.1)	9 (28.1)	15 (46.9)	6 (18.8)	1 (3.1)	32 (100)
Neutropenia	0 (0)	14 (43.8)	4 (12.5)	3 (9.4)	0 (0)	21 (65.6)
Anemia	1 (3.1)	7 (21.9)	11 (34.4)	0 (0)	0 (0)	19 (59.4)
Thrombocytopenia	4 (12.5)	3 (9.4)	6 (18.8)	6 (18.8)	0 (0)	19 (59.4)
Fatigue	5 (15.6)	7 (21.9)	6 (18.8)	0 (0)	0 (0)	18 (56.3)
Nausea	4 (12.5)	5 (15.6)	0 (0)	0 (0)	0 (0)	9 (28.1)
Diarrhea	3 (9.4)	2 (6.3)	2 (6.3)	0 (0)	0 (0)	7 (21.9)
Vomiting	5 (15.6)	2 (6.3)	0 (0)	0 (0)	0 (0)	7 (21.9)
Edema limbs	4 (12.5)	2 (6.3)	0 (0)	0 (0)	0 (0)	6 (18.8)
Neuropathy	5 (15.6)	1 (3.1)	0 (0)	0 (0)	0 (0)	6 (18.8)
Pneumonitis	1 (3.1)	4 (12.5)	0 (0)	0 (0)	1 (3.1)	6 (18.8)
Anorexia	0 (0)	4 (12.5)	1 (3.1)	0 (0)	0 (0)	5 (15.6)
Arthralgias	2 (6.3)	2 (6.3)	0 (0)	0 (0)	0 (0)	4 (12.5)
Weight loss	2 (6.3)	2 (6.3)	0 (0)	0 (0)	0 (0)	4 (12.5)
Elevated transaminases	1 (3.1)	2 (6.3)	0 (0)	0 (0)	0 (0)	3 (9.4)
Dehydration	0 (0)	1 (3.1)	1 (3.1)	0 (0)	0 (0)	2 (6.3)
Electrolyte imbalance	1 (3.1)	0 (0)	1 (3.1)	0 (0)	0 (0)	2 (6.3)
Febrile neutropenia	0 (0)	0 (0)	2 (6.3)	0 (0)	0 (0)	2 (6.3)
Fever	2 (6.3)	0 (0)	0 (0)	0 (0)	0 (0)	2 (6.3)
Hypotension	0 (0)	2 (6.3)	0 (0)	0 (0)	0 (0)	2 (6.3)
Nasal congestion	1 (3.1)	0 (0)	1 (3.1)	0 (0)	0 (0)	2 (6.3)
Rash	1 (3.1)	1 (3.1)	0 (0)	0 (0)	0 (0)	2 (6.3)

**Table 3:** Pneumonitis cases.

Patient	Prior immune- therapy	Prior radiation to the chest	Received steroids	Recovered	Discontinued protocol treatment^	Received subsequent standard treatment
1	No	Yes	Yes	Yes	Yes	Yes
2	No	No	Yes	Yes	No	Yes
3	Yes	No	Yes	Yes	No	Yes
4	Yes	Yes	Yes	Yes	No	No
5	Yes	No	No	Yes	No	Yes
6*	Yes	Yes	No	No	Yes	No

*Patient elected to pursue hospice, ^all patients discontinued gemcitabine.

### Subsequent therapy

Out of 32, twenty-nine subjects developed RECIST progression. Twenty-one (72.4%) patients progressed systemically, six (1.75%) progressed in the brain while two progressed both systemically and in the CNS. Those who progressed in the CNS only, three develop new brain metastases while remaining three develop disease progression in the previously treated lesions. Nineteen patients received further systemic therapy with a median of 2 lines of treatment (range 1–4). Nine patients enrolled in another clinical trial. Four patients received radiation following progression, 3 extracranial and 1 intracranial. One patient underwent surgical resection of a brain metastasis.

## Discussion

This study met its primary endpoint of an improvement in ORR to 28.1%, showcasing the activity of the combination of gemcitabine and nab-paclitaxel in patients with relapsed SCLC. In addition, we report an overall survival of 9.3 months, which is promising in a patient population with several poor prognostic factors, including 12% with an ECOG performance score of 2, over half of the population with a platinum-resistant/refractory disease, and 40% with treated brain metastases at the time of enrollment.

While there have been slight improvements in outcomes for patients with relapsed SCLC in the past few years, the ORR in our patient population is comparable to ORR for treatments for relapsed SCLC, including treatments that are incorporated in the National Cancer Consortium Network guidelines (**Table 4**). In addition to an improvement in ORR, our study showed a median OS of 9.3 months, which is comparable to commonly used treatments for relapsed SCLC, including lurbinectedin and topotecan (**Table 4**) (10, 11, 34, 35). While we recognize that these studies cannot be directly compared, we believe that these results are encouraging. Our findings highlight the need for future studies to compare gemcitabine and nab-paclitaxel with current available treatments for relapsed SCLC.

**Table 4:** Comparison of treatment options mentioned in NCCN guidelines for ES-SCLC in second-line or beyond except for tarlatamab.

Treatment	Trial type	Study size (*n)*	RR (%)	PFS (mos)	OS (mos)	G3+ NTP (%)	Chemo-resistant/refractory (%)	CNS involvement (%)
Lurbinectedin, doxorubicin (12)	RCT	307	31.6	4.0	8.6	37	32	15
Topotecan (10)	RCT	107	24.3	3.3	6.3	89	NR	11
CAV (10)	RCT	104	18.3	3.1	6.2	72	NR	24
Topotecan (21)	RCT	71	7.0	4.1	6.5	61	58	NR
Tarlatamab (22)	10 mg cohort 100 mg cohort	100 88	40% 32%	4.9 3.9	14.3 NE	0 5	22 26	23 36
Lurbinectedin (11)	Single arm	105	35.2	3.5	9.3	46	43	4
Nivolumab (23, 24)	Single arm	98	10.0	1.4	5.7	0	31	NR
Pembrolizumab (25)	Single arm	83	19.3	2.0	7.7	0	NR	15.7
Temozolomide (26)	Single arm	64	22.0	1.6	5.8	5.0	25	38.0
Bendamustine (27)	Single arm	50	26.0	4.0	4.8	4.0	42	NR
Gemcitabine (13)	Single arm	42	11.9	NR	7.1	27	43	NR
Gemcitabine (14)	Single arm	38	13.2	2.0	4.3	18	NR	NR
Docetaxel (28)	Single arm	34	25.0	NR	NR	NR	NR	NR
Gemcitabine, nab-paclitaxel (current)	Single arm	32	28.1	2.9	9.3	22	53	41
Oral etoposide (29)	Single arm	26	23.0	NR	NR	NR	NR	NR
Vinorelbine (30)	Single arm	26	16.0	NR	NR	NR	NR	NR
Temozolomide (31)	Single arm	25	12.0	1.8	5.8	8	36	NR
Paclitaxel (16)	Single arm	24	29.0	2.0	3.3	42	NR	NR
Vinorelbine (30)	Single arm	26	16.0	NR	NR	NR	NR	NR
Pembrolizumab (6)	Single arm	24	33.3	1.9	9.7	0	NR	12.5
Oral etoposide (32)	Single arm	22	45.5	NR	3.5	NR	NR	NR
Paclitaxel (15)	Single arm	21	23.8	NR	5.8	64	48	NR
Irinotecan (33)	Single arm	15	47.0	0.8*	6.2*	33	NR	20

RCT, randomized clinical trial; G3+, ≥grade 3; NTP, neutropenia; NR, not reported; NE, not estimable.

*Described in weeks or days.

Treatments for relapsed SCLC are commonly limited by hematologic toxicities. For example, topotecan has been reported to cause grade 4 neutropenia in 70% of patients. While lurbinectedin causes less neutropenia, grade 3 or 4 neutropenia has been reported to occur in 46% of patients. We found that gemcitabine and nab-paclitaxel led to less grade 3 or 4 neutropenia (22%; **Table 4**). In addition, 6.3% of patients developed febrile neutropenia in our study, which is comparable to febrile neutropenia in 5% of patients treated with lurbinectedin and lower than the reported 3–28% of patients on topotecan. Patients enrolled in lurbinectedin monotherapy and combinational therapies received prophylactic growth factor support while 62.5% of patients in this trial required growth factor support (11). Finally, only 6.3% of patients in this trial required treatment discontinuation due to adverse events, indicating that the combination is relatively tolerable.

In this study, nearly 19% of patients developed pneumonitis, of which 1 required treatment discontinuation and 1 was grade 5. Pneumonitis has been reported with this combination in pancreatic cancer but occurred less frequently (4%) in patients receiving this combination (18). The patient population included in this study may have increased risk factors for pneumonitis, such as a history of smoking (current or former = 100%), underlying lung disease (COPD 100%), previous receipt of radiation to the chest (62%), and previous exposure to ICI (56%). We could not find any other known predisposing factor for pneumonitis in these cases. A similar observation was made in a study that recruited non-small cell lung cancer patients who received the combination of gemcitabine and nab-paclitaxel, which led to grade 2–3 pneumonitis in 11% of patients (36).

Recent developments and an improved understanding of SCLC disease mechanisms are leading biomarker-driven drug development in SCLC. In particular, clinical trials are underway evaluating therapies targeted at delta-like ligand-3 through a bispecific antibody (tarlatamab) and targeting seizure-related homolog 6 (SEZ6) protein through an antibody-drug conjugate (22, 37). In addition, there has been interest in poly (ADP ribose) polymerase inhibitors in patients with Shlafen 11 expression (38). As more innovative therapies emerge, further developments of the combination of gemcitabine and nab-paclitaxel can be considered accordingly.

### Study limitations

Limitations of this study include a single-institution study including only a small number of White patients, which may make our findings less generalizable. In addition, outcomes in patients with relapsed SCLC have improved modestly since the design and conduct of this study, which may make comparisons to our trial more difficult. As biomarker-based therapies emerge, sequencing of treatments for relapsed/refractory ES-SCLC is yet to be defined.

## Conclusions

In patients with SCLC, relapse is inevitable and is associated with a poor prognosis. Treatment options for patients with relapsed SCLC remain limited. The combination of gemcitabine and nab-paclitaxel may be an effective and safe treatment option albeit with higher incidence of hematologic toxicities if utilized after topotecan or lurbinectedin. Further studies are needed to validate the therapeutic value of this regimen in larger patient populations and directly compare this combination with other approved options.

## Data availability statement

The original contributions presented in the study are included in the article/supplementary material, further inquiries can be directed to the corresponding author/s.

## Ethics statement

The studies involving humans were approved by Hawk IRB, University of Iowa, Iowa City, IA 52246. The studies were conducted in accordance with the local legislation and institutional requirements. The participants provided their written informed consent to participate in this study.

## Author contributions

MB: Visualization, Validation, Data curation, Writing – review & editing, Writing – original draft, Investigation. GS: Methodology, Funding acquisition, Conceptualization, Writing – review & editing, Writing – original draft. WZ: Resources, Investigation, Writing – review & editing. SM: Writing – original draft, Visualization, Formal analysis, Data curation, Writing – review & editing. GZ: Validation, Methodology, Writing – review & editing, Formal analysis. AK: Writing – original draft, Data curation, Writing – review & editing. JZ: Resources, Methodology, Investigation, Writing – review & editing, Writing – original draft. TA-H: Conceptualization, Writing – review & editing, Writing – original draft, Resources, Methodology, Investigation. GC: Funding acquisition, Writing – review & editing, Resources, Methodology, Conceptualization. MF: Visualization, Validation, Supervision, Project administration, Investigation, Formal analysis, Data curation, Writing – review & editing, Writing – original draft, Resources, Methodology, Funding acquisition, Conceptualization.
